# Editorial: NLRP3 activation and regulation in innate immune responses

**DOI:** 10.3389/fimmu.2023.1171138

**Published:** 2023-03-08

**Authors:** Alessandra Mortellaro

**Affiliations:** San Raffaele Telethon Institute for Gene Therapy (SR-Tiget), IRCCS San Raffaele Scientific Institute, Milan, Italy

**Keywords:** NLRP3, inflammasome, pyroptosis, chronic inflammation, IL-1 (interleukin-1)

The NLRP3 inflammasome is a multiprotein complex involved in the regulation of the immune system and inflammation (1). It comprises three main proteins: NLRP3 (Nod-like receptor protein 3), ASC (apoptosis-associated speck-like protein containing a caspase recruitment domain), and caspase-1. When activated, NLRP3 recruits the adaptor protein ASC and caspase-1 to form the inflammasome. Caspase-1 cleaves pro-inflammatory cytokines, such as interleukin-1β (IL-1β) and interleukin-18 (IL-18), into their active forms, which help recruit and activate immune cells to remove the stressor. Gasdermin D (GSDMD) is another inflammasome effector protein cleaved by caspase-1 downstream of inflammasome activation Magnani et al. The cleavage of GSDMD releases its N-terminal domain, which can form pores in the cell membrane, leading to a type of programmed cell death called pyroptosis.

Excessive or chronic activation of the N LRP3 inflammasome can contribute to various inflammatory diseases, including autoinflammatory diseases, such as cryopyrin-associated periodic syndrome (CAPS), autoimmune diseases, cancer, cardiovascular diseases, and neurological disorders (Alehashemi and Goldbach-Mansky) (2, 3). Understanding how NLRP3 inflammasome works and how its activation is regulated is important for developing new ways to prevent or treat inflammatory diseases.

This Research Topic brings together original articles related to the identification of new danger signals and pathways that activate the NLRP3 inflammasome. Moreover, some contributions highlight new mechanisms of inflammasome activation in human primary cells, like macrophages and neutrophils. Furthermore, some studies implicate chronic NLRP3 inflammasome activation in the pathogenesis of diseases, like chronic and acute pulmonary inflammation, spinal cord injury and liver fibrosis.

The NLRP3 inflammasome is known to sense a wide range of stimuli, including pathogen-associated molecular patterns (PAMPs) and danger-associated molecular patterns (DAMPs). PAMPs are molecular patterns associated with pathogens, such as bacteria, viruses, and fungi; on the other hand, DAMPs are molecules released from damaged or dying cells and tissues. These can include ATP, uric acid, or cholesterol crystals. Original articles in this Research Topic identify new endogenous activators and repressors of the NLRP3 inflammasome. The finding that the internalization of membrane attack complex (MAC), a component of the complement system, drives NLRP3 inflammasome activation and IL-1β secretion is highlighted by Diaz-del-Olmo et al. Upon endocytosis, the colocalization of MAC with NLRP3-ASC proteins leads to the assembly of the NLRP3 inflammasome. Wisitpongpun et al. describe that oleamide, a natural oleic acid derivative, can induce NLRP3 inflammasome-mediated release of IL-1β, potentiating the polarization of primary human macrophages toward a pro-inflammatory M1 phenotype. Kienes et al. report a new mechanism of how NLRP11, a member of the Nod-like receptor family, inhibits the activation of the NLRP3 inflammasome. They identified the ATP-dependent RNA helicase DDX3X as a protein binding to NLRP11. Therefore, by sequestering DDX3X, NLRP11 negatively regulates NLRP3-mediated caspase-1 activation, thereby, limiting inflammasome activation.

The tight regulation of the NLRP3 inflammasome pathway is crucial for maintaining proper immune function and preventing excessive inflammation. The mechanisms of NLRP3 inflammasome activation may depend on the specific context and cell type. While priming is a well-established step for NLRP3 inflammasome activation in many cell types, including murine macrophages, Gritsenko et al. report that priming might be dispensable for NLRP3 inflammasome activation in human monocytes *in vitro*. When human monocytes are treated with nigericin, it induces K+ and Cl- efflux, which triggers the assembly of the NLRP3 inflammasome complex, leading to IL-1β and IL-18 release. Inflammasome activation is also peculiar in neutrophils in several ways. Inflammasome activation in neutrophils is often associated with NETosis, a specialized form of programmed cell death in which neutrophils release net-like structures called neutrophil extracellular traps (NETs). Münzer et al. show that, under sterile conditions, neutrophils can assemble and activate the NLRP3 inflammasome with the support of the enzyme PAD4 (peptidyl arginine deiminase 4), which is involved in chromatin decondensation and NET formation. In addition, Keitelman et al. reveal that serine proteases support caspase-1 in the processing and secretion of IL-1β in human neutrophils *via* the autophagic pathway. Son et al. present another key feature of inflammasome activation in neutrophils. When exposed to a milieu enriched in DAMPs, neutrophils are resistant to pyroptosis and mitochondrial depolarization in response to a NLRP3 inflammasome activator. In contrast, macrophages are desensitized *via* a mechanism leading to mitochondrial depolarization and pyroptosis. Based on these results, they propose that neutrophils are the primary source of IL-1β released in DAMP-rich inflammatory districts.

On this Research Topic, some articles emphasize the contribution of excessive and chronic inflammasome activation in the pathogenesis of various inflammatory diseases. Huot-Marchand et al.demonstrate that the NLRP3 inflammasome and GSDMD are key players in pulmonary inflammation and remodeling upon acute or sub-chronic mouse exposure to cigarette smoke. Li et al. report that a 4-benzene-indol derivative ameliorates LPS-induced sepsis-related acute lung injury by disrupting NLRP3-NEK7 interaction and the subsequent inflammasome assembly and activation. Wang et al. focus on the role of the inflammasome-induced IL-1 release in the development and progression of spinal cord injury. Inhibition of caspase-4 (involved in NLRP3 inflammasome activation) and IL-1-mediated pathway attenuate inflammation and promote repair of the injured spinal cord by inhibiting NF-kB signaling, NLRP3 inflammasome, and GSDMD-mediated pyroptosis. The potential role of inflammasomes and pyroptosis in relation to liver fibrosis is reviewed in Gan et al. They particularly focus on the effect of inflammasome activation in various liver cells (i.e., hepatocytes, cholangiocytes, hepatic stellate cells, hepatic macrophages, and liver sinusoidal endothelial cells) and how the pharmacological treatment of inflammasomes can be exploited as a potential strategy for attenuating liver fibrosis.

To summarize, this Research Topic, with a variety of articles, has provided important new insights into the activation mechanisms of the NLRP3 inflammasome and its link with a number of diseases and conditions.

## Author contributions

The author confirms being the sole contributor of this work and has approved it for publication.
